# Validation of QTLs for Resistance to Pre-Harvest Sprouting in a Panel of European Wheat Cultivars

**DOI:** 10.3390/plants14091342

**Published:** 2025-04-29

**Authors:** Bruno Rajković, Ana Lovrić, Marko Maričević, Dario Novoselović, Hrvoje Šarčević

**Affiliations:** 1 Bc Institute for Breeding and Production of Field Crops, Rugvica, Dugoselska 7, 10370 Dugo Selo, Croatia; bruno.rajkovic@bc-institut.hr (B.R.); ana.lovric@bc-institut.hr (A.L.); marko.maricevic@bc-institut.hr (M.M.); 2 Centre of Excellence for Biodiversity and Molecular Plant Breeding (CroP-BioDiv), Svetošimunska Cesta 25, 10000 Zagreb, Croatia; dario.novoselovic@poljinos.hr; 3 Department of Cereal Breeding and Genetics, Agricultural Institute Osijek, Južno Predgrađe 17, 31000 Osijek, Croatia; 4 Faculty of Agriculture, University of Zagreb, Svetošimunska Cesta 25, 10000 Zagreb, Croatia

**Keywords:** wheat, pre-harvest sprouting, dormancy, KASP markers

## Abstract

Pre-harvest sprouting (PHS) of wheat poses a major challenge to global food security due to its negative impact on grain yield and quality. In the present study, we conducted the validation of previously published markers or functional markers associated with PHS resistance in a panel of 200 wheat cultivars adapted to Southeastern European conditions. In field experiments conducted in four environments in Croatia, the germination index (GI) was assessed, and significant genetic, environmental, and genotype–environment interactions were detected. The broad-sense heritability for GI was high (0.86), confirming the predominant role of genetic factors in determining PHS resistance. Twenty-two polymorphic SNP markers were analyzed for their effects on GI, of which nine markers from chromosomes 3A, 3B, 4A, 5A, and 7B showed significant genotypic effects across environments, especially TaMKK3-A and wsnp_Ex_rep_c66324_64493429. In addition, nine marker combinations were identified, which showed significant differences in GI between allele combinations. Overall, this study elucidates the genetic basis of PHS resistance in wheat cultivars adapted to the agro-climatic conditions of Southeast Europe and provides insights for marker-assisted breeding strategies to improve PHS resistance.

## 1. Introduction

Wheat (*Triticum aestivum* L.) is one of the three most widely cultivated cereals globally, alongside rice and maize. It is grown on more than 225 million hectares, with a total production of 776.7 million tons [1], feeding approximately 40% of the world’s population and providing more than 20% of the calories and proteins consumed by humans [2]. Therefore, maintaining high and stable yields while ensuring optimal end-use quality is a critical objective in global wheat production. Both yield and quality can be adversely affected by numerous biotic and abiotic stresses, including pre-harvest sprouting (PHS)—defined as the germination of grain in the spike before harvest, typically triggered by rainfall [3].

PHS leads to significant reductions in both grain yield and end-use quality [4,5], with global economic losses resulting from PHS estimated to exceed one billion dollars annually [6].

Genetic resistance to PHS, considered the most effective long-term strategy to mitigate its impact, is a quantitative trait governed by complex interactions among morphological and physiological traits of the spike and grain, along with strong environmental influence [7,8,9]. The phenotypic evaluation of PHS resistance under field conditions is challenging due to its environmental dependency. Moreover, quantitative trait loci (QTL) associated with PHS resistance have been identified on all 21 wheat chromosomes, reflecting the polygenic nature of this trait [7,10,11,12,13,14,15]. This complexity makes the breeding for PHS resistance particularly demanding and confirms PHS resistance as one of the most multigenic traits in wheat [16].

While many studies have investigated the mechanisms of PHS resistance, recent research has increasingly focused on seed dormancy (SD)—recognized as the primary genetic mechanism conferring PHS resistance [3,5]. Seed dormancy is defined as the inability of a viable seed to germinate under favorable conditions [8,17]. In Croatia and the rest of Southeastern (SE) Europe, PHS-inducing conditions do not occur consistently across growing seasons, necessitating laboratory-based phenotyping, which is time-consuming, destructive, and labor-intensive. Furthermore, the phenotyping of traits related to grain characteristics requires plants to reach physiological maturity. This complicates early selection in breeding programs, particularly when a large number of lines need to be assessed within a short time window between harvest and sowing [18].

Conventional breeding approaches are limited in improving PHS resistance due to its polygenic nature. Therefore, marker-assisted selection (MAS) depends heavily on the availability of tightly linked and validated QTL-associated markers or functional markers [3,19,20]. Recent studies have further demonstrated that these candidate genes play distinct yet complementary roles in seed dormancy regulation. *TaPM19-A1* and *TaPM19-A2* exhibit ABA-inducible expression during seed maturation and are differentially expressed due to an 18-bp promoter deletion associated with reduced dormancy [21]. The MAP kinase gene *TaMKK3-A*, located within the same *Phs-A1* interval, contains a functional C660A SNP that alters kinase activity and has been validated as the causal gene underlying natural variation in dormancy across global germplasm [16,22]. In parallel, the *TaPHS1* (*TaMFT-3A*) gene on chromosome 3AS was identified as a major determinant of seed dormancy and pre-harvest sprouting resistance. Initial work by Nakamura et al. [23] identified a −222 C > T SNP in the promoter region, which was associated with seed dormancy. Subsequent fine mapping and map-based cloning by Liu et al. [24] revealed two coding region mutations at +646 G > A and +666 C > T, which disrupt splicing and translation, leading to reduced dormancy in susceptible genotypes.

A novel 33-bp insertion/deletion at −194 bp in the promoter was later reported and functionally validated by Jiang et al. [25], with the favorable allele showing a strong association with improved PHS resistance. Additionally, Liu et al. [26] identified a 732 C > T SNP in the 3′ untranslated region, significantly associated with the sprouting phenotype in Chinese cultivars. To enable efficient MAS, multiple diagnostic KASP markers have been developed targeting these functional mutations, including PHS-646 and PHS-666 for the coding region SNPs [27], SNP_222 for the promoter SNP, and MFT-A2 for the −194 bp InDel [25].

Other regulatory loci include the *TaSdr-B1* and *TaSdr-A1* genes on chromosomes 2B and 2A, respectively, whose expression is positively associated with dormancy, and whose favorable alleles (*TaSdr-B1a*, *TaSdr-A1a*) have been widely identified in Chinese germplasm [14,28,29]. The transcription factor gene *TaVp-1B*, a wheat homolog of maize *Viviparous-1*, also plays a role in maintaining seed dormancy; specific haplotypes such as *Vp-1Bb*, *Vp-1Bc*, *and Vp-1Bf* are strongly associated with reduced sprouting [30,31,32,33].

The *Tamyb10-A1* gene, located on chromosome 3AL and classically associated with red pericarp pigmentation, functions as a Myb-type transcription factor regulating the flavonoid biosynthesis pathway in the seed coat. Its role in seed dormancy has been attributed to the accumulation of proanthocyanidin-derived compounds that physically and chemically suppress germination, with mutant alleles leading to reduced dormancy and premature sprouting [34,35]. More recently, a related D-genome homeolog, *Myb10-D*, was identified and functionally characterized as the causal gene underlying the *qPHS.sicau-3D* locus in a synthetic wheat population. Despite being a D-genome gene, *Myb10-D* was physically mapped to a 2.7 Mb presence-absence variation (PAV) region on chromosome 3DL (572.2–574.9 Mb), due to a structural variation specific to the SHW-L1 line [36]. Unlike Tamyb10-A1, which modulates dormancy indirectly via seed coat pigmentation, *Myb10-D* acts directly on the abscisic acid (ABA) biosynthesis pathway by transcriptionally activating *NCED*, a key ABA biosynthetic gene, thereby enhancing dormancy and reducing pre-harvest sprouting [36,37]. These findings illustrate functional divergence among *Myb10* homoeologs and underscore the complex regulatory network controlling PHS resistance in wheat.

In addition to the *Myb10* gene family, a novel gene, *TaPI4K-2A*, has been recently identified as a regulator of pre-harvest sprouting resistance through GWAS in a diverse wheat panel [38]. This gene encodes a phosphatidylinositol 4-kinase implicatated in ABA signaling, and two functional polymorphisms were associated with variation in seed dormancy; an InDel at −1359 bp in the promoter region, which disrupts an ABA-responsive cis-element (ABRE), and a non-synonymous SNP at +718 bp (Ala240Thr) in the coding sequence. The favorable haplotype (Hap1), carrying the ABRE-intact promoter and the Thr-240 allele, was linked to higher gene expression, stronger ABA sensitivity, and enhanced dormancy. Functional validation through overexpression and RNAi lines confirmed the role of *TaPI4K-2A* in modulating PHS resistance, making it a promising candidate for MAS.

Most previous QTL and GWAS studies have focused on non-European wheat germplasm. However, recent efforts have expanded to European material. For example, Albrecht et al. [39] conducted a GWAS on 124 elite European winter wheat lines and identified 22 QTLs, including stable loci on 3AS and 4AL associated with PHS resistance. More recently, Dallinger et al. [10] validated the *Phs-A1* locus on 4A, highlighting *TaPM19* and *TaMKK3-A* as candidate genes, and additionally reported novel QTLs on chromosomes 1A and 5B in a study involving 1373 winter wheat lines from 14 European countries and Canada.

This work builds upon earlier findings by Shorinola et al. [40], who mapped *Phs-A1* to a narrow genetic interval in UK germplasm and provided strong evidence that *TaMKK3-A*, rather than *PM19*, is the primary gene underlying dormancy variation at this locus.

Although these studies significantly contributed to our understanding of PHS resistance in European wheat, there remains a lack of data for wheat germplasm adapted to SE Europe, where unique agro-climatic constraints and breeding goals require targeted validation. This underscores the importance of evaluating the relevance and utility of known PHS resistance loci and markers within breeding materials from SE Europe.

Nevertheless, despite the identification of numerous QTLs for PHS resistance, relatively few studies have undertaken marker validation across genetically diverse germplasm. Most such efforts have focused on validating functional markers, as reported in some previous studies [2,9,14,24,27,30,31,41], as well as in the recent work by Wang et al. [33].

In the present study, we performed the validation of 38 previously published markers or functional markers associated with PHS resistance using a panel of 200 wheat cultivars adapted to SE European conditions. The specific objectives were (i) to evaluate the PHS resistance of the studied cultivars across four environments and (ii) to investigate the allelic effects of tested markers on resistance to PHS.

## 2. Results

### 2.1. Phenotypic Evaluation of PHS Resistance

The combined ANOVA for 200 wheat genotypes (Table S1) across four environments showed significant variation among genotypes (G), environments (E), and a significant G × E interaction for the germination index (GI). Genotypic, environmental, and G × E interaction variance accounted for 46, 22, and 27% of the total phenotypic variance, respectively, with an estimated broad sense heritability of 0.86 (Table 1).

Mean GI (Table 2) was lowest for Zagreb 2020 (0.19) and highest for Zagreb 2018 (0.50). However, a wide range of GI values across genotypes was observed in all four environments, with several genotypes showing similar levels of PHS resistance to the resistant check RL 4137 (Table S2).

### 2.2. Genotyping

**Figure 1:**
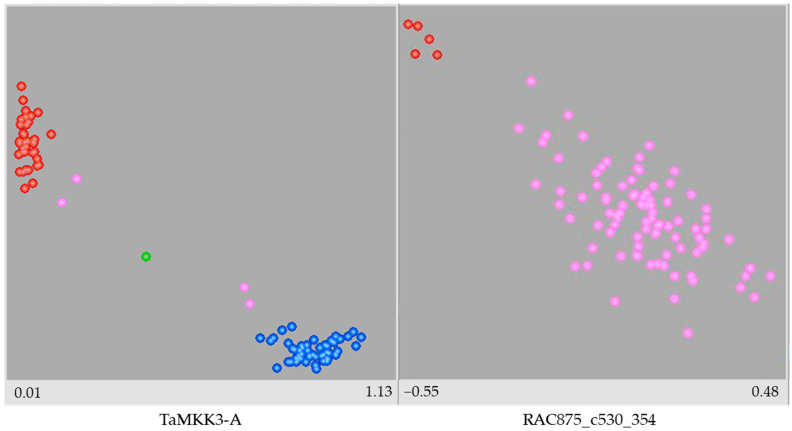
Scatter plots of genotyping results for TaMKK3-A and RAC875_c530_354 KASP markers generated by LGC SNP Viewer. Blue dots stand for homozygous allele group 1; red dots stand for homozygous allele group 2; green dots stand for heterozygous alleles; pink dots stand for no call due to inconsistent signal or failed amplification.

The results of the KASP assay for 38 markers (36 SNP and two indel markers) and 200 wheat genotypes are shown in Table S3. An example of genotyping results for two KASP markers visualized as scatter plots is shown in Figure 1. The KASP marker TaMKK3-A produced a clear and well-separated scatter plot with two distinct homozygous clusters indicating reliable genotype discrimination. In contrast, marker RAC875_c530_354 failed to produce clear genotype clusters. As can be seen in the scatter plot, almost all data points of RAC875_c530_354 are represented by pink dots, indicating samples that were not assigned a genotyping call due to an inconsistent signal or failed amplification.

For the markers TaMFT_1617R and TaPHS1-666, KASP primers were designed in the present study for a DNA strand complementary to the original DNA strand for which polymorphism was reported in previous studies. Therefore, the SNP polymorphisms for these markers are shown side by side in Table S3 as the polymorphism obtained in the present study and as the expected polymorphism from the original studies. To be comparable with the earlier studies, the expected polymorphism for these markers was used in the data analysis.

**Figure 2:**
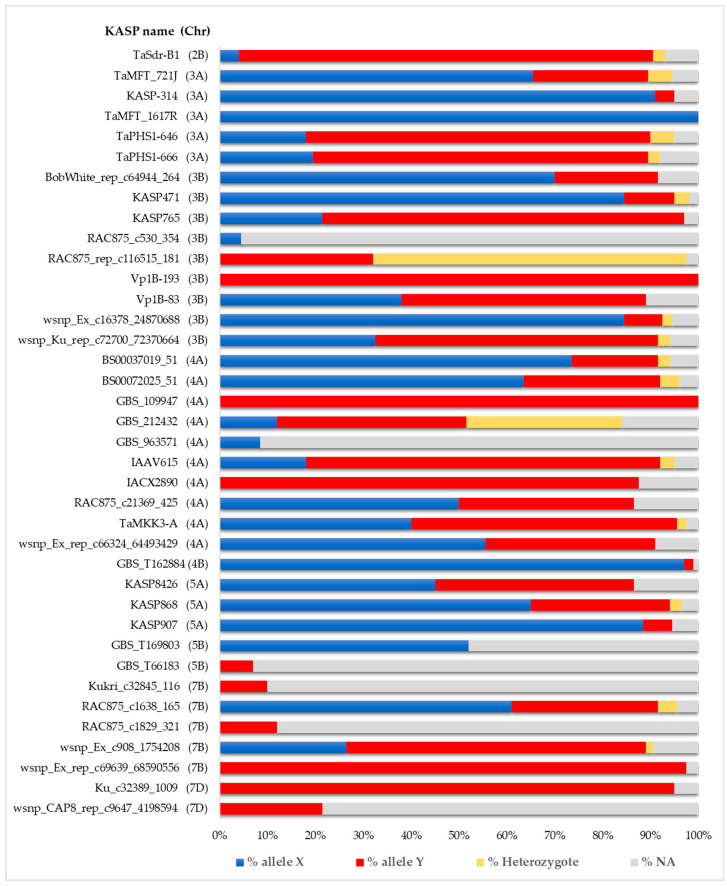
Proportions of homozygous and heterozygous genotypes and the proportion of missing data (NA).

The summary of the results of the KASP assay for 38 markers with the proportion of homozygous and heterozygous genotypes and the proportion of missing data is shown in Figure 2. Most KASP markers (26 out of 38) had less than 10% missing data (ranging from 0 to 9.5%), five markers had between 11 and 16%, and six markers had between 70.5 and 95.5% missing data.

The last six markers (GBS_963571, GBS_T66183, Kukri_c32845_116, RAC875_c1829_321, wsnp_CAP8_rep_c9647_4198594, RAC875_c530_354) were excluded from further statistical analysis due to the extremely high rate of missing data. Of the 38 SNP markers, 22 showed zero heterozygosity, 14 had heterozygosity levels below 5%, while two markers GBS_212432 (32.5%) and RAC875_rep_c116515_181 (65.5%) exhibited very high heterozygosity rates. Therefore, the two markers were also excluded from further statistical analyses. Of the remaining 30 markers, seven were excluded because they were monomorphic (GBS_109947, IACX2890, Ku_c32389_1009, TaMFT_1617R, Vp1B-193, GBS_T169803, wsnp_Ex_rep_c69639_68590556), and one marker (GBS_T162884) was excluded due to a minor allele frequency of only 2%. In summary, 22 polymorphic SNP markers located on various chromosomes were analyzed for their effects on the germination index, considering only homozygous genotypes.

### 2.3. Allelic Effect of KASP Markers on Germination Index

The allele frequencies at 22 KASP marker loci and their effect on GI are shown in Table S4. Marker effects were estimated by simple linear regression, performed both within individual environments and across environments, using the corresponding genotypic BLUP values. Minor allele frequencies at 22 marker loci ranged from as low as 4%, observed for the functional marker TaSdr-B1 (chromosome 2A), to 0.43 for the functional marker Vp1B−83.

Nine markers had a significant effect on GI in at least two out of four environments and can be considered stable (Table 3). Two markers, TaMKK3-A and wsnp_Ex_rep_c66324_64493429, both located on chromosome 4A, exhibited significant allelic effects across all four environments. Five additional markers were significant in three environments: the functional markers TaPHS1-646 and TaPHS1-666 from chromosome 3A, KASP765 from 3B, and BS00037019_51 and IAAV615 from 4A. Two other markers showed a significant effect in two environments: BS00072025_51 from chromosome 4A and wsnp_Ex_c908_1754208 from 7B. Among them, the marker combination TaPHS1-646/TaPHS1-666 from chromosome 3A and BS00037019_51/IAAV615 from chromosome 4A were in complete linkage disequilibrium and only two haplotypes were observed for the two combinations (Tables S3 and S4). The effect of haplotype for these two marker combinations is also shown in Table S4. The marker wsnp_Ex_c908_1754208 (7B) showed a significant effect on GI in two out of four environments (ZG2020 and OS2020), but no significant effect was observed across all environments (Table S4). Another marker, BobWhite_rep_c64944_264 (3B), also showed a significant effect on GI; however, the same allele had an opposite effect in ZG2018 compared to ZG2020 and OS2020.

Two markers Vp1B−83 and RAC875_c21369_425 showed a significant effect on GI in only one environment. Notably, for the functional marker Vp1B−83, the allele Vp1Bc, which carries an 83-bp deletion, was associated with increased GI. This result is contrary to expectations, as the same allele had previously been identified in a genotype classified as tolerant in another study.

A summary of the single-marker analysis for stable markers showing a significant reduction in GI in at least two environments is presented in Table 3. In the table, the effect of a favorable (“tolerant”) allele is expressed as the reduction in mean GI relative to the mean of the unfavorable (“susceptible”) allele class for the same marker. Additionally, the proportion of phenotypic variance explained by each marker, as determined by simple linear regression, is also provided.

The mean reduction in GI across different environments due to the effect of the tolerant (low-GI) allele ranged from 18% for TaPHS1-646 to 33% for TaMKK3-A. The proportion of phenotypic variance explained by these two markers was 3.1 and 17.5%, respectively. However, in individual environments, some markers showed substantially stronger effects, with GI reductions of up to 51% and 25% of the phenotypic variance explained, as observed for TaMKK3-A in the OS2020 environment.

Due to the possible linkage between the markers and a certain degree of collinearity, Akaike’s Information Criteria (AIC) were calculated to evaluate all possible subsets of the multiple regression models simultaneously and to determine the best model (best marker combination) that excludes the GI. Multiple linear regression analysis was performed including all stable individual markers (Table 3), except TaPHS1-666 and BS00037019_51, which were in complete linkage disequilibrium with TaPHS1-646 and IAAV615, respectively (Table S3). The results of the 10 best multiple linear regression models with the effects of the tolerant alleles of the markers in each model are shown in Table 4.

The best of the 10 models with the lowest AIC value contained only the functional marker TaMKK3-A and explained 18% of the phenotypic variance for GI. In this model, the tolerant allele reduced GI by 0.14. The following models (2 to 10) combined two to three markers, but all included TaMKK3-A, whose tolerant allele reduced GI by 0.13 to 0.17. The effect of the tolerant alleles at other marker loci was significantly lower compared to TaMKK3-A (−0.01 to −0.04) and the effect of the tolerant allele at marker locus BS00072025_51 (4A) even has a negative effect in combination with other markers, consistently increasing the GI by 0.04 to 0.05. None of the nine marker combinations was superior to TaMKK3-A alone, not even with regard to the proportion of explained phenotypic variance, which is only slightly higher for these combinations (19 to 20%).

The effects of haplotypes within nine marker combinations (models 2 to 10) on GI are shown in Table 5. In models containing three markers, seven out of eight possible haplotypes were observed. In models with two markers, all four possible haplotypes were present, except for model 8, which lacked one haplotype.

However, in all three-marker combinations (models 3, 9, and 10) and one two-marker combination (model 2), a unique haplotype appears that belongs to the cultivar Alixan (Table S3). These haplotypes were excluded from the Tukey–Kramer test when comparing haplotype means. In all models, there were significant differences between the mean GIs of the haplotypes. The GI of the best haplotype ranged from 0.22 (model 3) to 0.28 (model 5). The best haplotypes in all models contain tolerant alleles (highlighted in blue) of all markers with the exception of the marker BS00072025_51, whose susceptible allele is contained in the best haplotypes. In addition, all second-ranked haplotypes (whose GI in some cases is not significantly different from the best haplotype in a combination) contain the tolerant allele of TaMKK3-A, which is not the case for other markers represented by susceptible alleles (models 4 to 9).

**Figure 3:**
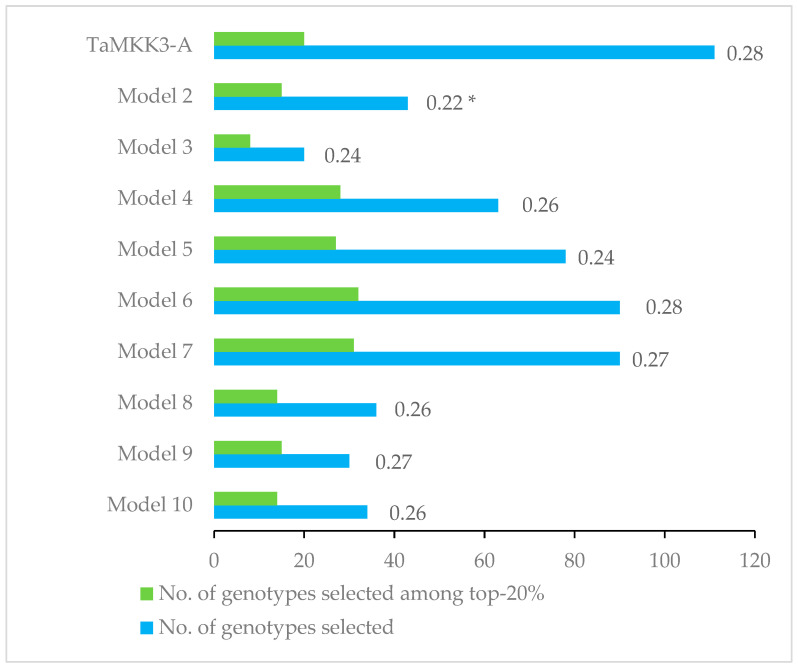
Mean germination index (GI) across environments for the tolerant allele class of TaMKK3-A and the best haplotypes of nine marker combinations (models 2 to 10). * Mean GI significantly different from GI of TaMKK3-A tolerant allele class.

To compare the efficiency of selection based on the best single marker TaMKK3-A (model 1) with nine marker combinations (models 2 to 10), we compared the GIs of the tolerant allele class of TaMKK3-A with the GIs of the best haplotypes using the *t*-test. We also compared the number of genotypes selected from the 10 models belonging to the top-20% (most dormant) genotypes (Figure 3). In model 2, the GI of the best haplotype was significantly lower than that of the TaMKK3-A-tolerant class. However, no significant differences were found for the GIs of the remaining models compared to TaMKK3-A. Despite the lower GI, model 2 identified only 15 highly dormant genotypes, compared to 20 selected using TaMKK3-A. In contrast, in models 4 to 7, the number of highly dormant genotypes selected was significantly higher (27 to 32) than in TaMKK3-A selection. These four models (4 to 7) all contain TaMKK3-A and the markers wsnp_Ex_rep_c66324_64493429, wsnp_Ex_c908_1754208, KASP765, and TaPHS1-646, respectively.

## 3. Discussion

This study aimed to evaluate the effectiveness of previously reported genetic markers associated with PHS resistance in a diverse panel of winter wheat cultivars. The panel comprised 200 genotypes, carefully assembled to represent a genetically and geographically diverse set of registered cultivars adapted to the agroecological conditions of SE Europe. A targeted validation approach was applied using single-marker regression, multiple regression models, and haplotype analysis. This enabled the identification of informative markers applicable to locally adapted germplasm and provided practical insights for implementing marker-assisted selection strategies in regional breeding programs. The cultivars were phenotyped for PHS resistance across four environments using germination tests performed on harvest-ripe grains, from which the germination index (GI) was calculated. A wide range of GI values was observed among the tested genotypes, indicating substantial genetic variation for PHS resistance within the panel, and several PHS-resistant cultivars were identified (Table S2). Combined analysis of variance (ANOVA) across environments revealed that genotype (G), environment (E), and genotype by environment interaction (G × E) accounted for 46%, 22%, and 27% of the total phenotypic variance, respectively. The estimated broad-sense heritability (H^2^) of 0.86 further confirms the predominant role of genetic factors in determining PHS resistance. These results are consistent with previous studies reporting broad-sense heritability estimates for PHS resistance ranging from 0.66 to 0.96 [14,15,33,39,42,43]. Building upon the observed high heritability and genetic variation for PHS resistance, the performance of individual markers was further examined across environments. Of the 22 polymorphic SNP markers analyzed in the present study, nine markers—including the functional markers TaPHS1-646, TaPHS1-666, and TaMKK3-A—were identified as stable, as they consistently showed a significant effect on reducing the germination index (GI) in at least two out of the four test environments (Table 3 and Table S4). These markers are therefore considered promising candidates for marker-assisted selection targeting stable PHS resistance. The average reduction in GI associated with the favorable alleles of stable markers ranged from 17% to 33% across environments. Notably, the effect of certain markers in specific environments was substantially stronger. For example, TaMKK3-A exhibited the highest effect, reducing GI by 51% in the environment OS2020.

Among all markers analyzed in the present study, TaMKK3-A, located on chromosome 4AL, showed the strongest and most stable effect on pre-harvest sprouting (PHS) resistance. The favorable allele reduced the germination index (GI) by an average of 33% across environments, while its closely linked SNP wsnp_Ex_rep_c66324_64493429 showed a comparable effect (30%). These were the only two markers that showed a significant reduction of GI in all four test environments, confirming the robustness of the *TaMKK3-A* locus as a major contributor to PHS resistance. The functional role of *TaMKK3-A* was first demonstrated by Torada et al. [22], who identified a causal SNP (C660A) resulting in an amino acid substitution (N220K), which enhances seed dormancy. This finding was further validated by Shorinola et al. [16], who demonstrated a perfect association between allelic variation at *TaMKK3-A* and dormancy phenotype across 11 biparental mapping populations and a MAGIC population. They also genotyped two major germplasm panels: the Watkins collection of 804 landraces and the Gediflux panel of 457 modern European cultivars. While the favorable C allele was prevalent in landraces, its frequency in modern cultivars was notably lower (52%), reflecting a historical breeding preference for rapid and uniform germination. In the present study, the frequency of the dormant C allele was 58%, which is consistent with these trends. Further support for the utility of *TaMKK3-A* in diverse germplasm comes from studies in U.S. and Chinese breeding materials. Vetch et al. [9] observed a significant effect of *TaMKK3-A* in 21 U.S. winter wheat cultivars, and Li et al. [15] confirmed the presence and effect of *TaMKK3-A* within the *Phs-A1* QTL region using 15K SNP arrays in genome-wide linkage mapping. In addition, Martinez et al. [44] provided compelling evidence that *TaMKK3-A* is the causal gene underlying *Phs-A1*, and not the nearby *PM19-A1/A2* genes, which, although physically close, are functionally distinct. In contrast, Huang et al. [14] did not detect a statistically significant effect of *TaMKK3-A* on GI in their panel of 326 Chinese winter wheat cultivars, with differences between alleles being smaller than 2% across three growing seasons. The authors suggested that this lack of effect could be due to genetic background or near fixation of the favorable allele in their material.

The *TaPHS1* gene, also known as *TaMFT*, is one of the most thoroughly studied loci for PHS resistance in wheat. In the present study, two functional SNPs located at positions +646 and +666 within this locus were polymorphic, forming two distinct haplotypes: the favorable GA haplotype and the susceptible AT haplotype. Genotypes carrying the GA haplotype exhibited a significant reduction in the germination index (GI) of about 20% compared to those carrying the AT haplotype (Table 3). This finding is in agreement with previous studies conducted on U.S., South African, and Chinese germplasm [9,14,24,41,42], which also identified the +646/+666 GA haplotype as a stable and effective marker combination for PHS resistance. However, compared to our study, Liu et al. [24] and Shao et al. [42] reported a much greater reduction in sprouting rate by 84 and 38% in 167 and 82 U.S. cultivars, respectively, possessing the GA haplotype. The haplotype frequencies observed in the present study (~0.8 GA: 0.2 AT) closely mirror those reported by Liu et al. [24] and Shao et al. [42], who found frequencies of 0.80: 0.20 and 0.77: 0.23, respectively, in panels of U.S. winter wheats. Although only GA and AT haplotypes were detected in our panel, additional recombinant haplotypes (GT and AA) have been identified at low frequencies in South African and Chinese germplasm [14,41]. In contrast, the SNP located at position −222 (KASP marker TaMFT_1617R) was monomorphic in our panel, with all genotypes carrying the unfavorable T allele. This observation is consistent with findings from Wamalwa et al. [45], who also reported fixation of the T allele in 239 wheat cultivars and breeding lines from Kenya and Ethiopia. Previous studies have reported inconsistent effects of this SNP: it showed no significant association with PHS in Liu et al. [24] but was found to have a weaker yet statistically significant effect in Shao et al. [42]. Additionally, Wang et al. [2] reported that the C allele at −222 reduced the average sprouting rate by approximately 30% compared to the T allele across three experiments in Chinese wheat accessions. Two additional markers within the *TaPHS1* region, TaMFT_721J [27,46] and KASP-314 [42], were also evaluated in the present study but did not show significant associations with GI. While other polymorphisms have been proposed at the *TaPHS1* locus, such as indels or haplotype-specific markers, their utility remains largely confined to specific regional germplasm and has not been validated broadly. Dong et al. [47] reported a significant effect of *TaPHS1* locus on PHS resistance in 344 Chinese wheat cultivars using a PCR marker showing a 12-bp indel variation. However, the effect of this newly discovered polymorphism still needs to be validated in wheat germplasm from outside of China. Altogether, the results of this study and previous literature highlight the +646/+666 GA haplotype as the most consistent and reliable marker combination for PHS resistance at the *TaPHS1* locus. Nonetheless, as demonstrated by Fakthongphan et al. [48], substantial phenotypic variation may still be observed in populations fixed for the favorable haplotype, suggesting that additional loci—possibly including those on chromosome 3A—contribute to the polygenic nature of PHS resistance.

Among the markers that did not show a consistent effect across environments in the present study, *Vp-1B3*—a gene known to regulate seed dormancy through abscisic acid (ABA) signaling—was not significantly associated with variation in the germination index (GI). Specifically, no significant differences were observed between genotypes carrying the wild-type, non-dormant Vp-1Ba allele and those carrying the Vp-1Bc allele, which contains an 83-bp deletion and was previously linked to enhanced seed dormancy. These findings are in agreement with previous studies by Vetch et al. [9] and Huang et al. [14], who also reported a lack of significant association between Vp-1B alleles and PHS resistance in U.S. and Chinese winter wheat germplasm, respectively. On the contrary, earlier research by Yang et al. [49] and Chang et al. [31] demonstrated a strong association between the Vp-1Bc allele and increased PHS resistance, particularly in Chinese wheat accessions.

In the current panel of 200 SE European winter wheat cultivars, the frequencies of the Vp-1Ba and Vp-1Bc alleles were 43% and 57%, respectively. These frequencies are comparable to those reported by Xia et al. [32] in European germplasm (with Vp-1Bb being rare at only 1%) and by Huang et al. [14] in Chinese wheat (40% Vp-1Ba, 3% Vp-1Bb, 57% Vp-1Bc). Similarly, Wamalva et al. [45] found the Vp-1Bc allele to be the predominant variant (~80%) among 239 wheat cultivars and breeding lines from Kenya and Ethiopia, suggesting that this allele is widespread across diverse germplasm pools. Recently, three SNPs were identified within the coding sequence (CDS) of the *TaVP1-B* gene in wheat [33]. The alleles at the three SNP positions were in complete LD, forming only two haplotypes that showed significant association with PHS resistance in a panel of 304 Chinese wheat cultivars. However, the effect of this newly discovered polymorphism on PHS resistance remains to be confirmed in European germplasm. Altogether, while earlier studies reported strong effects of Vp-1B on PHS resistance, particularly in Asian germplasm, results from the present study and other genetically distinct panels suggest that the predictive value of Vp-1B3 alleles may vary across populations.

The *TaSdr* gene family, comprising *TaSdr-A1*, *TaSdr-B1*, and *TaSdr-D1*, has been reported to be involved in regulating seed dormancy and tolerance to pre-harvest sprouting (PHS) in wheat. In the present study, only TaSdr-B1 was included in the marker panel, and the frequency of the favorable A allele—associated with higher dormancy—was found to be very low (4%) among 200 European winter wheat cultivars. As a result, no significant difference in the germination index (GI) was observed between genotypes carrying the alternative alleles. This is consistent with findings by Zhang et al. [28], who reported that the low-GI-associated TaSdr-B1a allele was not detected in cultivars from Germany, Romania, Russia, Ukraine, and Serbia. In contrast, the favorable allele was common in cultivars from Japan, Australia, Argentina, and southern regions of China, such as the Yangtze Valley and Southwest Winter Wheat Regions. On the other hand, Huang et al. [14] observed a significant reduction in GI in 326 Chinese winter wheat cultivars carrying the TaSdr-B1a allele. Moreover, this allele exhibited a synergistic effect when combined with TaPHS1-Hap1, resulting in a further reduction of GI in genotypes carrying both favorable variants (AC1 group). These findings suggest that combining advantageous alleles at these loci can enhance resistance to pre-harvest sprouting.

In addition to single-marker effects, in the present study, we also evaluated the explanatory power of multiple-marker regression models and compared haplotype combinations (Table 4 and Table 5). The strongest individual marker, TaMKK3-A, explained 18% of the phenotypic variance, while the best-performing multiple-marker models, which combined TaMKK3-A with one or two additional SNPs such as BS00072025_51 or KASP765, explained 20% of the phenotypic variance (Table 4). This modest increase in the proportion of phenotypic variance explained suggests that additional loci may contribute additively, but their effects appear limited in the genetic background of this panel. In addition, the effect of the tolerant alleles at other marker loci was much smaller than that of the tolerant allele of marker TaMKK3-A, suggesting that *TaMKK3-A* is a major gene controlling PHS resistance in the wheat panel studied. However, despite the similar mean GI found in the best haplotypes and in the tolerant allele class of TaMKK3-A alone, the selection of the best haplotypes resulted in a greater proportion of highly dormant genotypes compared to the single-marker selection.

Overall, the results of the present study and previous studies confirm the complex genetic background of PHS resistance in wheat, in which the effects of many underlying loci are population-specific.

## 4. Materials and Methods

### 4.1. Plant Materials and Field Experiments

The validation of KASP markers for pre-harvest sprouting (PHS) resistance in winter wheat was conducted on a panel of 200 genotypes adapted to the agroecological conditions of SE Europe. This panel encompassed released wheat cultivars sourced from diverse geographical origins, including Austria, Brazil, Germany, France, Italy, Croatia, Poland, Romania, Russia, Serbia, Turkey, Hungary, and the United Kingdom, along with the Canadian cultivar RL 4137, serving as a PHS-resistant control. Most of the cultivars included in the panel have been grown in the past (103 cultivars) or are still in commercial production (30 cultivars) in Croatia (Table S1), and, therefore, they are relevant for regional breeding programs. Field trials were conducted at two Croatian locations: Zagreb (during the 2017–2018, 2018–2019, and 2019–2020 growing seasons) and Osijek (in the 2019–2020 growing season). In all four trials, genotypes were arranged in a randomized complete block design (RCBD) with two replicates. Each experimental plot consisted of three rows, 120 cm in length, with 20 cm spacing between rows. One hundred seeds were sown per row, resulting in a plant density of 416 plants per m^2^. Field management followed standard agronomic practices, including soil preparation, fertilization, and pest control, to ensure optimal growing conditions for the winter wheat genotypes studied.

### 4.2. Sampling of Spikes and Quantification of Seed Dormancy (SD)

At physiological maturity (growth stage 92; [50]), 50 spikes per plot were harvested, with a grain moisture content of about 13% on a fresh weight basis. Pre-harvest sprouting (PHS) resistance was assessed using germination tests performed on manually threshed seeds. Forty surface-sterilized seeds per sample were placed crease-side down on filter paper laid on the bottom of Petri dishes and moistened with 5 mL of deionized water. The Petri dishes were incubated for six days in darkness at 20 °C in a growth chamber.

Germination was assessed by counting the number of germinated seeds after 3 and 6 days of incubation. Based on these observations, the germination index (GI) was calculated following the method originally proposed by Reddy et al. [51], with the modifications as described by Ikić et al. [52].

GI = ((6 × *n*_3_) + (3 × *n*_6_))/(6 × *N*)

In this calculation, *n*_3_ and *n*_6_ represent the number of seeds germinated on the third and sixth day of incubation, respectively; N denotes the total number of seeds tested; and the values 6 and 3 correspond to the weights assigned to seeds germinated on the third and sixth day, respectively. Notably, the germination index (GI) is inversely related to the degree of seed dormancy, with lower values indicating stronger dormancy.

### 4.3. DNA Extraction and KASP Genotyping

Samples of young leaves from five plants per genotype were collected using the BioArk™ Leaf Collection Kit (LGC, Biosearch Technologies™, Hoddesdon, UK). DNA extraction and genotyping were performed by LGC Biosearch Technologies™ (Hoddesdon, UK) as part of their all-inclusive genotyping service (KASP), following the manufacturer’s standard protocol [53]. A total of 38 Kompetitive Allele Specific PCR (KASP) markers associated with pre-harvest sprouting (PHS) resistance were used for genotyping all genotypes included in the field trials. These markers were selected based on their reported association with PHS resistance in previous studies and span key genomic regions on chromosomes 2B, 3A, 3B, 4A, 4B, 5A, 5B, 7A, 7B, and 7D. The assay design and primer synthesis were carried out by LGC Biosearch Technologies™ (Hoddesdon, UK). Marker sequences and corresponding primers were sourced from previously published literature [27,42,54,55,56,57]. The original source for each of the 38 KASP markers is listed in Table S5, enabling traceability of marker development and prior validation.

### 4.4. Statistical Analysis of Field Experiments and KASP Marker Validation

Statistical package Meta-R version 6.00 [58] was used to calculate best linear unbiased predictions (BLUPs) of GI for each genotype within each environment, based on the following model:

*Y_ik_* = *µ* + *g_i_* + *r_k_* + *ε_ik_*

where *Y_ik_* is the GI of the i-th genotype in the k-th replicate, *µ* is the grand mean, *g_i_* is the effect of the i-th genotype, *r_k_* is the effect of the k-th replicate, and *ε_ik_* is the error term.

The same package was used to calculate BLUPs, estimate the variance components, and calculate heritability for GI across environments using the model:

*Y_ijk_* = *µ* + *g_i_* + *e_j_* + *r_k_*(*e_j_*) + *g_i_* × *e_j_* + *ε_ijk_*

where *Y_ijk_* is the GI of the i-th genotype in the j-th environment and k-th replicate, *µ* is the grand mean, *g_i_* is the effect of the i-th genotype, *e_j_* is the effect of the j-th environment, *r_k_* is the effect of the k-th replicate, *g_i_* × *e_j_* is the effect of genotype × environment interaction, and *ε_ijk_* is the error term. All the effects in the model were considered random. Broad sense heritability was calculated using the following formula:

*h^2^* = *σ^2^_g_* / (*σ^2^_g_* + *σ^2^_g×e_* / *e* + *σ^2^_ε_* / *er*)

where *σ* ^2^_g_, *σ* ^2^*_g__×__e_*, and *σ* ^2^*_ε_* are genotype, genotype × environment interaction, and error variance components, respectively, and *e* and *r* are the number of environments and replicates, respectively.

Tukey’s Studentized Range (HSD) Test was used for testing differences between environmental means at *p* < 0.05. It was based on the following model:

*Y_ij_* = *µ* + *g_i_* + *e_j_* + *ε_ij_*

and was conducted using the GLM procedure of SAS version 9.4. [59], using BLUPs of GI for each genotype in each environment as input data. In the model, *Y_ij_* is the GI of the i-th genotype in the j-th environment; *µ* is the grand mean, *g_i_* is the effect of the i-th genotype, *e_j_* is the effect of the e-th environment, and *ε_ij_* is the error term.

### 4.5. Allelic Effect of KASP Markers on GI

To analyze the allelic effect of KASP markers on GI, simple linear regression was performed for each marker in individual environments as well as across environments using the corresponding genotypic BLUP values of GI. The effect of a favorable (“tolerant”) allele, in terms of reduction of mean GI compared to the unfavorable (“susceptible”) allelic class mean of the same marker, as an output of simple regression analysis, was also expressed relative to the “susceptible” allelic class mean.

Due to the possible linkage among markers and a certain degree of collinearity, Akaike’s Information Criterion (AIC) was employed to simultaneously evaluate all possible subsets of multiple regression models and identify the best-fitting model, representing the optimal combination of markers explaining variation in the germination index (GI). The model with the lowest AIC value was considered the best among all alternatives [60]. The multiple linear regression analysis was performed by including only stable single markers (showing significant effects on GI in at least two of four environments) as independent variables, and the genotypic BLUP values for GI across environments as dependent variables. Simple linear and multiple linear regression analyses were performed using statistical package SAS version 9.4 [59]. The GI means of the haplotypes within the 10 best models (marker combinations) were compared using the Tukey–Kramer test at *p* < 0.05 by fitting the following model of the GLM procedure of SAS version 9.4 [59]:

*Y_ijk_* = *μ* + *haplotype_i_* + *e_j_* + *g_k_* (*haplotype_i_*) + *haplotype_i_* × *e_j_* + *ε_ijk_*

where *Y_ijk_* is the GI of the k-th genotype within the i-th haplotype in j-th environment, *µ* is the grand mean, *e_j_* is the effect of the e-th environment, *haplotype_i_* is the effect of the i-th haplotype, and *ε_ijk_* is the error term. The genotypes within the haplotype class were considered random, whereas all other factors were considered fixed effects.

## 5. Conclusions

The present study provides a comprehensive validation of key markers for pre-harvest sprouting resistance in European winter wheat germplasm. Markers such as TaMKK3-A, TaPHS1-646, TaPHS1-666, and wsnp_Ex_rep_c66324_64493429 demonstrated consistent and significant effects on germination index reduction across multiple environments, underscoring their utility for marker-assisted selection (MAS). Conversely, the limited performance of markers such as Vp1B-83 and TaSdr-B1 in this panel emphasizes the importance of population-specific validation. The high broad-sense heritability observed for GI and the identification of stable QTL regions provide valuable tools for the development of wheat cultivars with improved PHS resistance adapted to the conditions in SE Europe. Further functional validation of candidate loci and exploration of new polymorphisms in underrepresented genomic regions may enhance future breeding strategies targeting PHS resistance.

## Figures and Tables

**Table 1:** ANOVA for the germination index (GI) of 200 wheat genotypes across four environments.

Source of Variation	Variance	Significance	% of the Total Variance	Heritability
Genotype (G)	0.031	<0.001	46	0.86
Environment (E)	0.015	<0.001	22	
G × E	0.018	<0.001	27	
Residual	0.003		5	

**Table 2:** Mean germination index (GI) of 200 wheat genotypes in four environments.

Year	Location	Environment	Mean GI ± SD	GI Range
2018	Zagreb	ZG2018	0.50 ± 0.25 a ^1^	0.03–0.98
2019	Zagreb	ZG2019	0.36 ± 0.21 b	0.02–0.97
2020	Osijek	OS020	0.33 ± 0.24 b	0.02–0.98
2020	Zagreb	ZG2020	0.19 ± 0.16 c	0.01–0.92
Mean			0.35 ± 0.18	0.04–0.89

SD, standard deviation; ^1^ environmental means of GI followed by the same letter are not significantly different at *p* < 0.05 according to Tukey’s HSD test.

**Table 3:** Allele frequency at nine marker loci and the effect of the tolerant (low-GI) allele on mean GI in four environments. The allele effect is expressed as the relative change in GI due to the effect of the tolerant allele compared to the susceptible allele (%) with the corresponding proportion of phenotypic variance explained by the marker (PVE).

SNP Marker Locus (Chr)	Freq (%)	Allele	Change of GI Due to the Effect of Tolerant Allele (%) Proportion of Phenotypic Variance Explained (PVE)				
			**ZG2018**	**ZG2019**	**ZG2020**	**OS2020**	**Across Environments**
TaPHS1-646 (3A)	20	A	ns	−23 (3.5)	−35 (4.6)	−22 (2.2)	−18 (3.1)
	80	**G ^1^**					
TaPHS1-666 (3A)	22	T	ns	−20 (3.5)	−31 (5.2)	−26 (3.0)	−20 (4.0)
	78	**A**					
KASP765 (3B)	22	A	ns	−21 (3.5)	−39 (8.6)	−38 (9.0)	−20 (4.3)
	78	**G**					
BS00037019_51 (4A)	80	C	−19 (2.7)	ns	−38 (3.7)	−46 (7.3)	−27 (5.2)
	20	**T**					
BS00072025_51 (4A)	69	A	ns	ns	−41 (7.0)	−37 (7.2)	−19 (3.4)
	31	**G**					
IAAV615 (4A)	80	G	−17 (2.3)	ns	−33 (3.2)	−41 (5.8)	−22 (4.4)
	20	**A**					
wsnp_Ex_rep_c66324_64493429 (4A)	61	C	−24 (6.5)	−25 (5.2)	−43 (10.0)	−49 (16.3)	−31 (12.7)
	39	**T**					
TaMKK3-A (4A)	42	T	−29 (11.1)	−18 (3.0)	−48 (17.2)	−51 (25)	−33 (17.5)
	58	**G**					
wsnp_Ex_c908_1754208 (7B)	30	C	ns	ns	−28 (3.9)	−23 (2.8)	ns
	70	**T**					

^1^ Tolerant (low-GI allele) is shown in bold.

**Table 4:** The top ten multiple regression models explaining the germination index (GI) of wheat cultivars in different environments. The associated chromosome for each marker is indicated in parentheses.

Model/Marker	BS00072025_51 (4A)	IAAV615 (4A)	KASP765 (3B)	TaMKK3-A (4A)	TaPHS1-646 (3A)	wsnp_Ex_c908_1754208 (7B)	wsnp_Ex_rep_c66324_64493429 (4A)	PVE	AIC
	Effect of tolerant allele on GI								
Model 1				−0.14				18	−553.13
Model 2	0.04			−0.17				19	−552.72
Model 3	0.05			−0.15			−0.04	20	−552.43
Model 4				−0.13			−0.03	19	−552.07
Model 5				−0.14		−0.02		19	−551.51
Model 6			−0.02	−0.14				19	−551.44
Model 7				−0.14	−0.01			19	−551.25
Model 8		−0.01		−0.14				18	−551.22
Model 9	0.04			−0.16		−0.02		20	−551.20
Model 10	0.05		−0.02	−0.16				20	−551.14

PVE—percent of phenotypic variance explained by the model; AIC—Akaike’s information criterion.

**Table 5:** Mean germination index (GI) across environments for haplotypes of nine marker combinations (models 2 to 10).

Model	Combination of KASP Markers	Haplotype							All
	No. of genotypes	43	56	78	1				178
2	BS00072025_51	A	G	A	G				
	TaMKK3-A	G	G	T	T				
	BLUP_GI LSMEAN	0.26 a	0.30 b	0.44 c	0.41				
	No. of genotypes	20	37	21	18	68	7	1	172
3	BS00072025_51	A	G	A	G	A	A	G	
	TaMKK3-A	G	G	G	G	T	T	T	
	wsnp_Ex_rep_c66324_64493429	T	T	C	C	C	T	C	
	BLUP_GI LSMEAN	0.22 a	0.26 ab	0.29 b	0.36 c	0.44 d	0.48 d	0.41	
	No. of genotypes	63	39	69	7				178
4	TaMKK3-A	G	G	T	T				
	wsnp_Ex_rep_c66324_64493429	T	C	C	T				
	BLUP_GI LSMEAN	0.24 a	0.33 b	0.44 c	0.48 c				
	No. of genotypes	78	25	42	28				173
5	TaMKK3-A	G	G	T	T				
	wsnp_Ex_c908_1754208	T	C	T	C				
	BLUP_GI LSMEAN	0.28 a	0.29 a	0.43 b	0.48 c				
	No. of genotypes	90	17	54	24				185
6	KASP765	G	A	G	A				
	TaMKK3-A	G	G	T	T				
	BLUP_GI LSMEAN	0.27 a	0.31 a	0.41 b	0.48 c				
	No. of genotypes	90	12	23	49				174
7	TaMKK3-A	G	G	T	T				
	TaPHS1-646	G	A	A	G				
	BLUP_GI LSMEAN	0.26 a	0.37 b	0.42 bc	0.44 c				
	No. of genotypes	36	64	79					179
8	IAAV615	A	G	G					
	TaMKK3-A	G	G	T					
	BLUP_GI LSMEAN	0.27 a	0.28 a	0.43 b					
	No. of genotypes	34	7	47	8	54	23	1	174
9	BS00072025_51	A	A	G	G	A	A	G	
	TaMKK3-A	G	G	G	G	T	T	T	
	KASP765	G	A	G	A	G	A	G	
	BLUP_GI LSMEAN	0.26 a	0.27 a	0.29 a	0.31 a	0.41 b	0.49 c	0.41	
	No. of genotypes	30	11	11	41	42	28	1	164
10	BS00072025_51	A	G	A	G	A	A	G	
	TaMKK3-A	G	G	G	G	T	T	T	
	wsnp_Ex_c908_1754208	T	T	C	T	T	C	T	
	BLUP_GI LSMEAN	0.24 a	0.24 ab	0.31 bc	0.32 c	0.43 d	0.48 e	0.41	

Tolerant and susceptible alleles are marked in blue and red, respectively; mean values of GI following the same letter are not significantly different according to the Tukey–Kramer test at *p* < 0.05.

## Data Availability

The original contributions presented in the study are included in the article/Supplementary Material; further inquiries can be directed to the corresponding authors.

## Supplementary Materials

The following supporting information can be downloaded at: https://www.mdpi.com/article/10.3390/plants14091342/s1. Table S1. Wheat cultivars used in the study: accession numbers, names, and countries of origin; Table S2: Best linear unbiased predictions (BLUPs) of the germination index (GI) for 200 wheat genotypes across four environments and their overall mean; Table S3: KASP genotyping results for 200 wheat cultivars evaluated at 38 previously reported marker loci associated with PHS resistance; Table S4: Comparison of allelic effects at 22 KASP markers on the germination index (GI) in four environments and across-environment average; Table S5: Detailed list of Kompetitive Allele Specific PCR (KASP) markers used for targeted validation of genomic regions associated with pre-harvest sprouting resistance in wheat, including gene or QTL context, variant type, observed alleles, full primer sequences, and original source references.

## Abbreviations

The following abbreviations are used in this manuscript:

PHS	pre-harvest sprouting
GI	germination index
SNP	single-nucleotide polymorphism
KASP	Kompetitive Allele Specific PCR
QTL	quantitative trait locus
SD	seed dormancy
SE	Southeastern Europe
MAS	marker-assisted selection
ABA	abscisic acid
MAP	mitogen-activated protein
PAV	presence/absence variation
NCED	nine-cis-epoxycarotenoid dioxygenase
GWAS	genome-wide association study
ABRE	abscisic acid-responsive element
RNAi	RNA interference
ANOVA	analysis of variance
SD	standard deviation
HSD	honestly significant difference
DNA	deoxyribonucleic acid
BLUP	best linear unbiased prediction
PVE	phenotypic variance explained
AIC	Akaike’s Information Criterion
MAGIC	multiparent advanced generation inter-cross
CDS	coding sequence
RCBD	randomized complete block design
PCR	polymerase chain reaction
SAS	statistical analysis system
